# Effects of tension on mitochondrial autophagy and osteogenic differentiation of periodontal ligament stem cells

**DOI:** 10.1111/cpr.13561

**Published:** 2023-10-13

**Authors:** Xiaoru Shao, Zhong Hu, Huiqin Su, Yuzhong Wang, Yunfeng Lin

**Affiliations:** ^1^ Department of Stomatology Affiliated Hospital of Jining Medical University Jining Shandong China; ^2^ College of TCM Shandong University of Traditional Chinese Medicine Jinan Shandong China; ^3^ State Key Laboratory of Oral Diseases, National Clinical Research Center for Oral Diseases, West China Hospital of Stomatology Sichuan University Chengdu Sichuan China; ^4^ Department of Neurology and Central Laboratory Affiliated Hospital of Jining Medical University Jining Shandong China; ^5^ Sichuan Provincial Engineering Research Center of Oral Biomaterials Chengdu Sichuan China

## Abstract

This study aimed to explore the osteogenic ability and mitochondrial autophagy of periodontal ligament stem cells (PDLSCs) under cyclic tensile stress (CTS). Primary PDLSCs were isolated from the periodontal membrane and cultured by passage. Alizarin red staining, alkaline phosphatase detection, reverse transcription polymerase chain reaction (RT‐PCR), and Western blotting were used to detect the osteogenic differentiation level of PDLSCs. Mitochondrial autophagy in PDLSCs after CTS was measured using a mitochondrial autophagy detection kit, and the expression levels of autophagy‐related proteins LC3B, LAMP1 and Beclin1 were measured using cellular immunofluorescence technology, RT‐PCR and Western blot. After applying CTS, the osteogenic differentiation ability of PDLSCs was significantly improved, and the expression of alkaline phosphatase on the surface of the cell membrane and the formation of calcium nodules in PDLSCs were significantly increased respectively. We also studied the relevant mechanism of action and found that applying CTS can promote the osteogenic differentiation of PDLSCs and is related to the activation of mitochondrial autophagy. This study provides new insights into the mechanism of increased osteogenic differentiation on the tension side of orthodontic teeth and provides new experimental evidence for the involvement of mitochondrial autophagy in the regulation of osteogenic differentiation.

## Graphical abstract

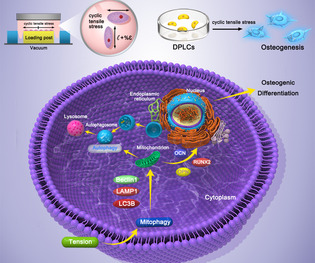

## 1 INTRODUCTION

Orthodontic tooth movement is the spatial displacement of teeth caused by the remodelling of the periodontal ligament and alveolar bone through the transfer of tensile force and static pressure to the alveolar bone from the periodontal ligament when a mechanical stimulus force is applied to the teeth.^1^, ^2^ Research has shown that the tension and static pressure generated during the orthodontic process increase bone formation on the tension side and bone absorption on the pressure side.^3^, ^4^ Periodontal ligament stem cells (PDLSCs) are mesenchymal stem cell/progenitor cells with multipotent differentiation and self‐renewal ability in the periodontal ligament.^5^, ^6^ PDLSCs express surface markers related to mesenchymal stem cells and specific biomarkers related to osteogenesis.^7^ In vitro studies have shown that PDLSCs can achieve osteogenic differentiation through specific induction medium. Numerous studies have shown that PDLSCs play a critical role in orthodontic tooth movement, bone reconstruction and maintaining the stability and regeneration of periodontal tissue.^8^, ^9^, ^10^, ^11^, ^12^ However, the mechanism of orthodontic force in PDLSC osteogenic differentiation needs further study.

Organelles are the important structural basis of cellular activity micronetworks. Mitochondria are the energy source and structural basis of stem cell osteogenic differentiation.^13^, ^14^ Therefore, maintaining the stability of the structure, quantity and function of mitochondria in mesenchymal stem cells is necessary for achieving stem cell osteogenic differentiation.^15^, ^16^, ^17^, ^18^ Mitochondrial autophagy, a major form of cell protection involved in controlling the quality and quantity of mitochondria, plays a critical part in achieving normal mitochondrial physiological functions and biological behaviours. Osteoblasts, odontoblasts and other vertebrate biomineralized host cells have been found to play a critical part in the collagen fibre and hydroxyapatite assembly process. These cells can accumulate calcium and hydrogen phosphate ions to form amorphous calcium phosphate, which is transported to lysosomes and transferred through mitochondrial autophagy. Finally, exocytosis participates in the body mineralization process. Regulation of intracellular mitochondrial content is a potential mechanism by which autophagy regulates the early directional differentiation of stem cells, and mitochondrial autophagy is a potential therapeutic target for bone‐related diseases.^19^, ^20^ However, few studies have focused on the changes in mitochondrial autophagy after disruption and its relationship with stem cell osteoblastic differentiation. Determining the mitochondrial autophagy process is of great significance for understanding the mechanism of orthodontic force on PDLSC osteogenesis.

This study aimed to investigate the changes in the osteogenic differentiation ability of PDLSCs after mechanical traction. We used the autophagy inhibitor chloroquine and inducer rapamycin to disrupt mitochondrial autophagy in PDLSCs and explored the changes in this process in cells after mechanical traction. The relationship between mitochondrial autophagy and the osteogenic differentiation of PDLSCs was also investigated. Overall, the findings further reveal the molecular biological mechanism of orthodontic tooth movement.

## 2 MATERIALS AND METHODS

### 2.1 Materials

The main reagents and kits used in this study were α‐MEM culture medium (Hyclone, Logan, UT, USA), penicillin–streptomycin (Hyclone, Logan, UT, USA), type I collagenase (Worthington, Lakewood, CO, USA), dispase (Sigma Aldrich, St. Louis, MO, USA), an RNA extraction kit (Fastagen, Shanghai, China), antibodies (Abcam, Cambridge, UK), a reverse transcription kit and a real‐time fluorescent quantitative polymerase chain reaction (RT‐PCR) kit (ABclonal, Boston, MA, USA), osteoblast induction medium (Cygen, Santa Clara, CA, USA) and an alkaline phosphatase detection kit (Beyond, Shanghai, China). Other reagents used are of analytical grade or better.

### 2.2 Isolation and culture of PDLSCs

According to the protocol approved by the Ethics Committee, PDLSCs were taken from premolars extracted from 18 to 35‐year‐old patients in the Oral and Maxillofacial Surgery Department of the Affiliated Hospital of Jining Medical College for orthodontic treatment. All the selected patients were informed about the procedure and signed informed consent documentation. PDLSCs were isolated and cultured as previously reported. Cells with the STRO‐1+/CD34−/CD45− immunophenotype were characterized using immunofluorescence, and their multipotency was established using adipogenic and osteogenic culture conditions. The PDLCs were cultured in conventional growth medium comprising α‐MEM, 100 U/mL penicillin/streptomycin and 10% FBS. At passages three to five, experimental group PDLSCs underwent uniaxial cyclic tensile stress (CTS); untreated PDLSCs were used as controls.

### 2.3 Application of CTS to PDLSCs

PDLSCs were plated in a flexible‐bottomed BioFlex culture plate (Flexcell) at a density of 3 × 10^5^ cells/well. Uniaxial CTS was applied (12% deformation and 6 cycles/min (5 s on and 5 s off)) to confluent PDLSCs for the indicated time points (24, 48 and 72 h) using a Flexcell® FX‐6000 TM Tension Unit (Flexcell). The control cells were maintained in an identical plate without CTS. The mitochondrial autophagy inhibitor chloroquine or the agonist rapamycin was added to cells 8 h before applying CTS for 48 h to investigate the effects of CTS on mitochondrial autophagy in PDLSCs and their ability to mediate osteogenic differentiation.

### 2.4 Induction of osteogenic differentiation of PDLSCs

An OriCell® Human Related Stem Cell Osteogenic Differentiation Kit was used to assess the effect of CTS on PDLSCs. PDLSCs were treated as described above, and then the culture medium was replaced with osteogenic induction medium to induce osteogenic differentiation. The osteogenic induction medium was replaced every 3 days. After 7 days of induced differentiation, RT‐PCR, Western blot and cellular immunofluorescence were used to investigate the osteogenesis‐related genes and proteins expression. After 7 and 21 days of induced differentiation, BCIP/tetrazolium nitrogen blue (NBT) alkaline phosphatase colour development kit was used to detect NBT formazan formation, and alizarin red was used to detect calcium nodule formation.

### 2.5 Reverse transcription polymerase chain reaction (RT‐PCR)

PDLSC groups were treated accordingly. After 7 days of culture, total RNA was extracted with a rapid extraction kit. Then ABScript III RT Master Mix for qPCR with gDNA remover was used to obtain stable cDNA. PCR was performed with 2× Universal SYBR Green Fast qPCR Mix following the manufacturer's protocol. Glyceraldehyde‐3‐phosphate dehydrogenase (GAPDH) was used as an internal reference to detect the changes in osteogenesis and autophagy‐related gene expression in PDLSCs after applying CTS. The primer sequences are shown in Table 1.

**SCHEME 1:**
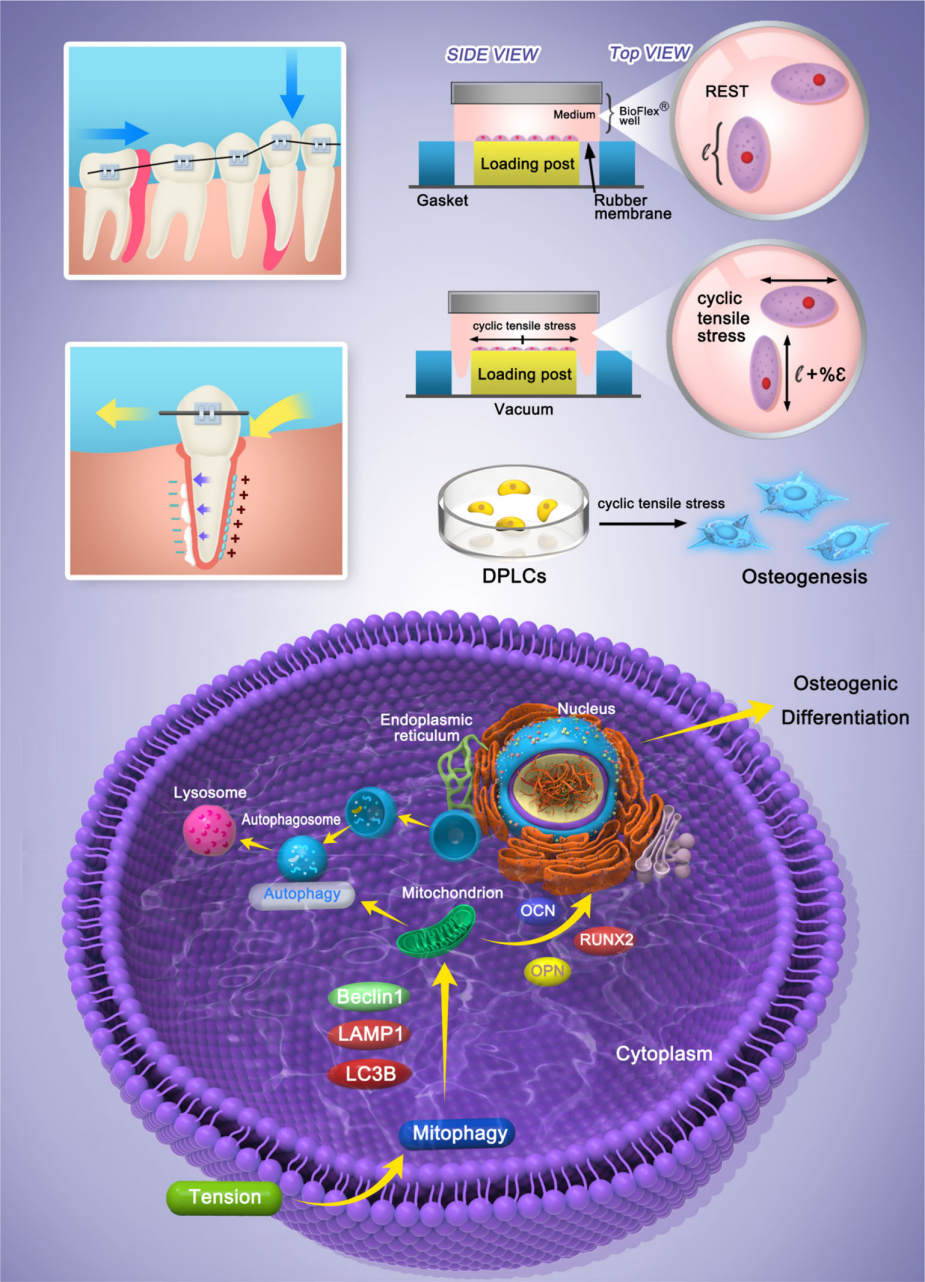
The effect of tension on mitochondrial autophagy and its ability to mediate osteogenic differentiation in PDLSCs.

**TABLE 1:** Primer sequences used to detect the relevant genes by RT‐PCR.

Genes	Primer (5′‐3′)	Sequence
*GAPDH*	Forward Reverse	AGGTCGGTGTAACGGATTTG TGTAGACCATGTAGTTGAGGTCA
*RUNX2*	Forward Reverse	TCCACACCATTAGGGACCATC TGCTAATGCTTCGTGTTTCCA
*OCN*	Forward Reverse	CACCGAGACACCATGAGAGC CTGCTTGGACACAAAGGCTGC
*OPN*	Forward Reverse	AGTGCAAATGTAATTGCGG CTGGTGCAAACGTAATTGC
*LC3B*	Forward Reverse	AAGGCGCTTACAGCTCAATG CTGGGAGGCATAGACCATGT
*Beclin 1*	Forward Reverse	ACCTCAGCCGAAGACTGAAG AACAGCGTTTGTAGTTCTGACA
*LAMP 1*	Forward Reverse	AAAGATGTGCTTCGAGATGTGT CACTTTGTCAGTTACCAACGTCA

### 2.6 Western blotting

The expression levels of bone differentiation and autophagy‐related proteins were assessed using Western blotting. Total proteins were extracted from PDLSCs using a total protein extraction kit. The protein samples were then isolated using SDS–PAGE and transferred to polyvinylidene fluoride (PVDF) membranes. The membranes were incubated with specific primary antibodies and visualized using an enhanced Bio‐Rad chemiluminescence detection system (GAPDH was used as the internal reference protein to normalize the target proteins expression). The primary antibodies used in this study were obtained from Abcam (Cambridge, MA, USA; RUNX2 (ab76956, 1:1000), OPN (ab214050, 1:1000), OCN (ab133612, 1:1000), LC3B (ab192890, 1:1000), LAMP1 (ab24170, 1:1000), Beclin1 (ab207612, 1:1000) and GAPDH (ab8245, 1:1000)).

### 2.7 Cellular immunofluorescence

PDLSC groups were treated accordingly, fixed, permeated and sealed using conventional methods. The cells were subsequently probed with the relevant primary antibodies at a dilution ratio of 1:200 overnight, followed by incubation with Alexa Fluor 594‐labelled secondary antibodies (Beyotime, Jiangsu, China), Phalloidin (Sigma Aldrich, St. Louis, MO, USA) and DAPI (Abcam, Cambridge, MA, USA) in the dark. Fluorescent images were captured using a fluorescence microscope (TCS SP8; Leica, Wetzlar, Germany).

### 2.8 Mitochondrial autophagy detection

Third‐generation periodontal stem cells were inoculated into the mechanical pull plate at a density of 5 × 10^7^ L^−1^. The experimental groups were divided according to the above methods. The induction group was treated with 10 μmol/L autophagy inducer rapamycin for 8 h, and the inhibition group was treated with 5 μmol/L autophagy inhibitor chloroquine for 8 h. Subsequently, the induction group and inhibition group medium was replaced with osteogenic medium for culture, treated with or without CTS for 48 h according to the group conditions, and stained using Mitophagy Detection Kit to assess mitochondrial autophagy. According to the instructions of the mitochondrial autophagy kit, the culture medium was aspirated and the culture plate was cleaned with sterile PBS. First, Mtphagy Dye working solution (1:250) was added and incubated overnight, and then complete osteogenic medium containing the mitochondrial autophagy inducer or inhibitor (without serum) was added and incubated overnight. Finally, Lyso Dye working solution (1:250) was added and incubated for 1 h, washed once with PBS and mitochondrial autophagy was observed by laser confocal microscopy. Mtphagy Dye is used to detect mitochondrial autophagy, and Lyso Dye is a lysosome dye. Under normal conditions, Mtphagy Dye binds to mitochondria and emits faint red fluorescence. Lyso Dye labels lysosomes with green fluorescence. When autophagy was induced by rapamycin, Mtphagy Dye produced strong fluorescence and colocalized with Lyso Dye to form yellow autophagic lysosomes.

### 2.9 Statistical analysis

Student's *t* test or one‐way ANOVA in GraphPad Prism version 8.0.2 (GraphPad Soft‐ware Inc., San Diego, CA, USA) were used for statistical analysis.

## 3 RESULTS

### 3.1 Isolation, culture and identification of human periodontal membrane stem cells

**FIGURE 1:**
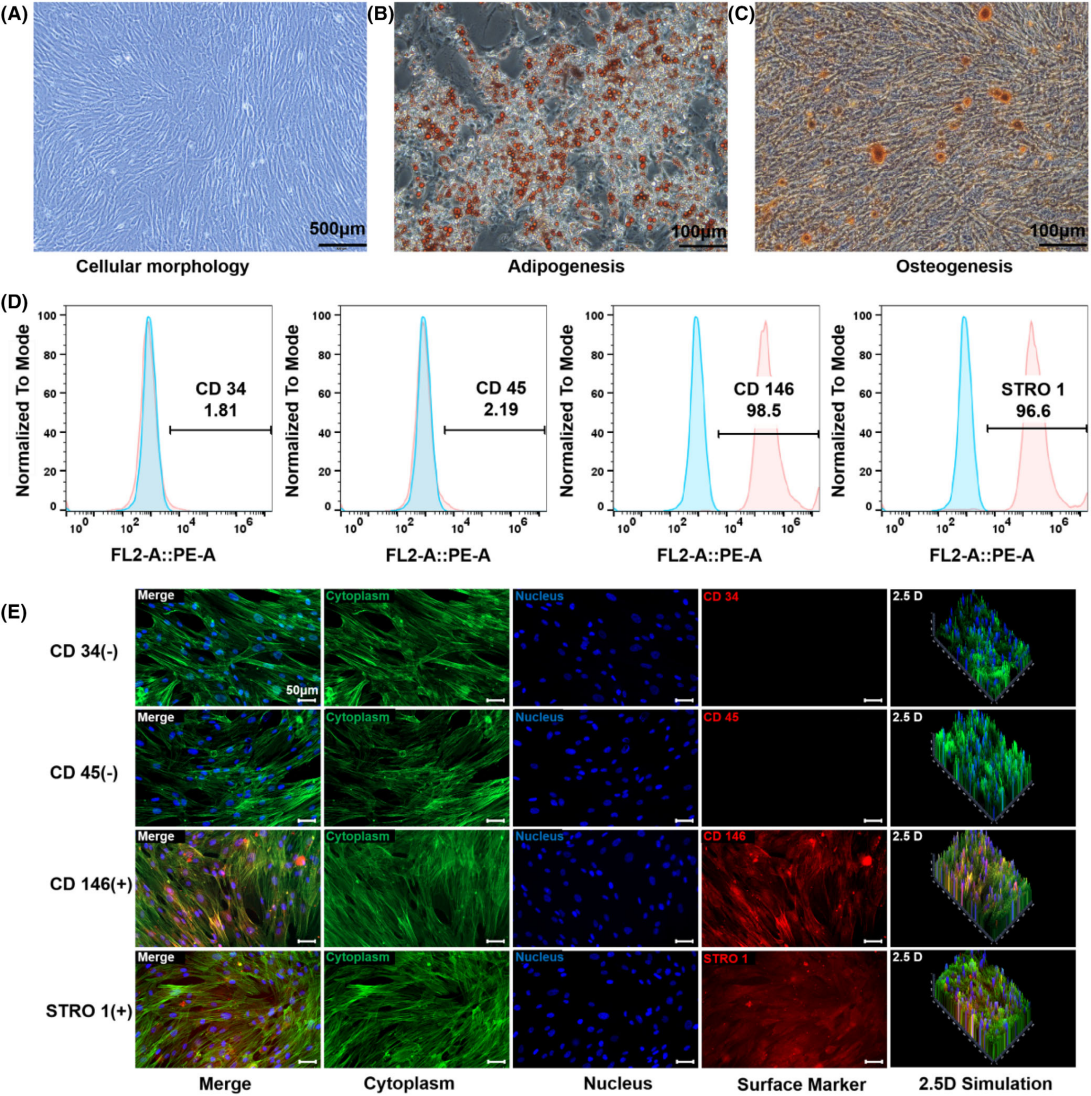
Acquisition and validation of periodontal membrane stem cells. (A) Primary PDLSCs were obtained through enzyme digestion (scale bars are 200 μm). B) Oil red O staining of PDLSCs after 14 days of adipogenic differentiation (scale bars are 100 μm). C) Alizarin red staining of PDLSCs after 21 days of osteogenic induction (scale bars are 100 μm). D) Flow cytometry showed that PDLSCs were positive for the MSC markers CD146 and STRO 1 but negative for the haematopoietic markers CD34 and CD45. E) Immunofluorescent micrographs showed that PDLSCs were positive for the MSC markers CD146 and STRO 1 but negative for the haematopoietic markers CD34 and CD45 (scale bars are 50 μm; cytoplasm: green; nucleus: blue; and surface marker: red).

The human periodontal stem cells isolated in this study adhered to the culture dish with fibroblast‐like morphology in circular colonies. Under the light microscope, the cells were full in shape, with clear cell outlines and nuclei and showed strong activity (Figure 1A). On the 14th day of osteogenesis induction, numerous red mineralized nodules were observed under the microscope after alizarin red staining (Figure 1C). Lipid formation was induced at 14 days as demonstrated using oil red O staining; numerous lipid droplets could be seen under a light microscope (Figure 1B). Flow cytometry (Figure 1D) showed that the cells were positive for CD 146 (98.5%) and STRO 1 (96.6%) and negative for CD 34 (1.81%) and CD 45 (2.19%). The immunofluorescence staining results (Figure 1E) were consistent with the flow cytometry results. The isolated cells in this group met the International Association for Cell Therapy standard for stem cell surface markers and had the potential to differentiate into bone and lipids, indicating that the isolated human periodontal membrane stem cells had the characteristics of mesenchymal stem cells.^21^

### 3.2 Effect of CTS on the osteogenic differentiation of PDLSCs

**FIGURE 2:**
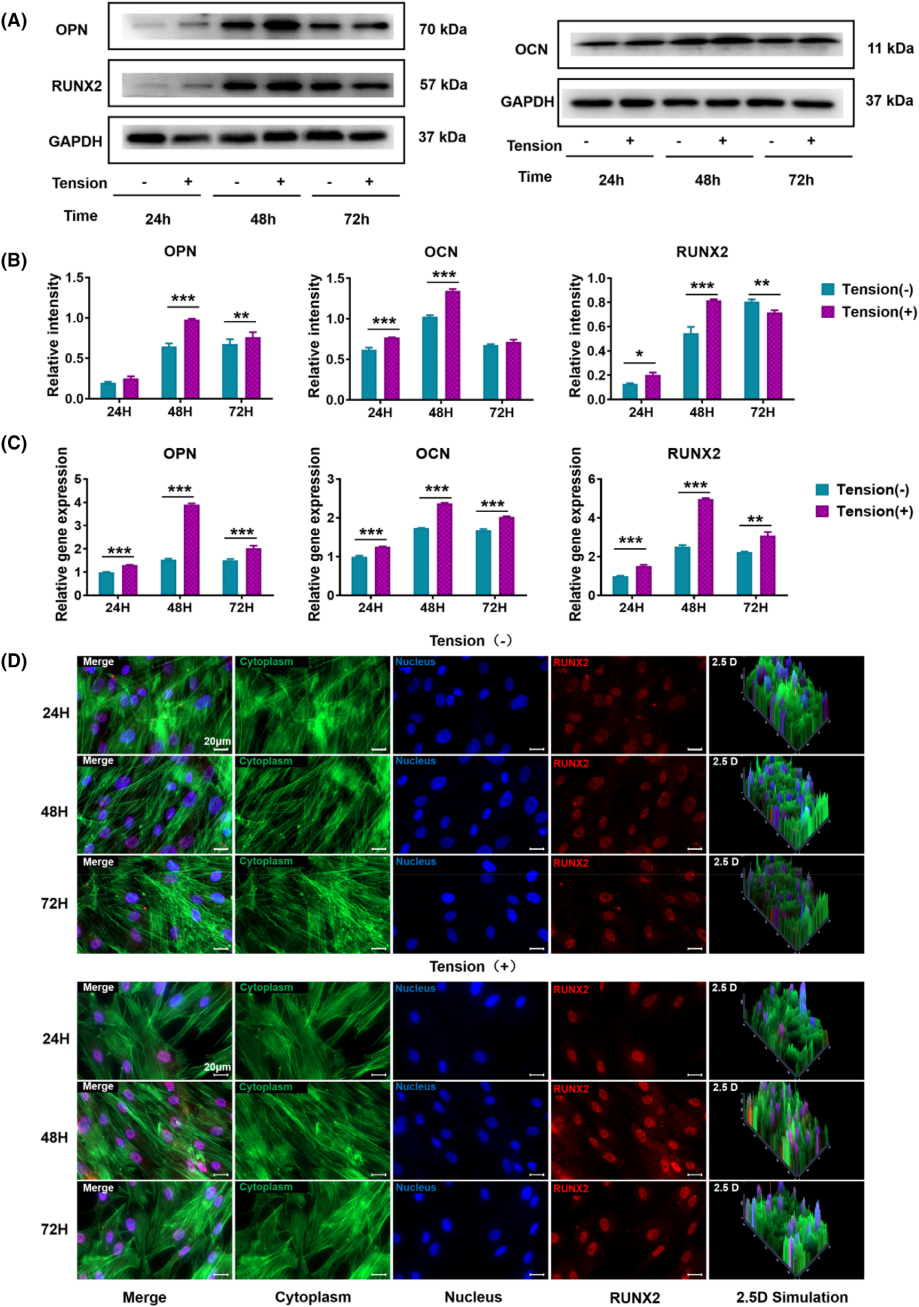
Effect of CTS on osteogenic differentiation of PDLSCs. (A) Western blotting was used to assess the expression levels of OCN, OPN, and RUNX2 in PDLSCs after 12, 24, and 48 h of CTS treatment (GAPDH was used as the internal control). (B) Quantification of OCN, OPN, and RUNX2 expression levels. GAPDH levels were set as the internal normalized control. Student's *t* test was used for the statistical analysis. Data are presented as the mean ± SD (*n* = 4). Statistical analysis: ** *p* < 0.01 and *** *p* < 0.001. (C) RT‐PCR was used to detect the gene expression levels of OCN, OPN, and RUNX2 in PDLSCs after 12, 24, and 48 h of CTS treatment. GAPDH levels were set as the internal normalized control. Student's *t* test was used for the statistical analysis. Data are presented as the mean ± SD (*n* = 4). Statistical analysis: ** *p* < 0.01 and *** *p* < 0.001. (E) Immunofluorescent micrographs of treated PDLSCs (scale bars are 50 μm; cytoplasm: green; nucleus: blue; and RUNX2: red).

PDLSCs were divided into three groups and treated with CTS for 12, 24 and 48 h to investigate the effect of mechanical tension on the osteogenic differentiation ability. The stress loading mode is a sinusoidal waveform with a deformation rate of 0–12% and a frequency of 0.1 Hz (Scheme). After 7 days of osteogenic induction, the expression levels of OCN, RUNX2 and OPN in the different treatment groups were detected by WB. Mechanical tension significantly promoted the expression of early and late markers of osteogenic differentiation in PDLSCs. The protein expression of osteogenic markers in PDLSCs was significantly upregulated after mechanical tension treatment, and these expression levels were most significant at 24 h (Figure 2A,B). Statistical analysis results showed that the expression of OPN, a marker of late osteogenic differentiation, was 1.27, 1.51 and 1.13 times higher than that of the control group at 12, 24 and 48 h, respectively. Additionally, the expression of RUNX2, a key transcription factor encoding osteogenic differentiation, was 1.47, 1.49 and 0.88 times higher than that of the control group at 12, 24 and 48 h, respectively. Furthermore, the expression of the bone tissue‐specific protein OCN was 1.23, 1.32 and 1.05 times higher than that of the control group at 12, 24 and 48 h, respectively. In addition, RT‐PCR was used to measure the expression of genes related to osteogenic differentiation when PDLSCs were subjected to mechanical tension at different times. Figure 2C showed that the gene expression levels of OCN, RUNX2, and OPN were significantly elevated after the mechanical traction of PDLSCs for 12, 24 and 48 h (OPN increased 1.31, 2.54 and 1.34 times, respectively; RUNX2 1.52, 1.98 and 1.38 times, respectively; and OCN 1.26, 1.36 and 1.21 times, respectively). These results suggest that mechanical tension can promote the osteogenic differentiation of PDLSCs, most significantly with a 24 h treatment, and mechanical tension can significantly upregulate the expressions of mRNA corresponding to osteogenic differentiation. In addition, we assessed the expression of proteins associated with osteogenic differentiation by immunofluorescence. The samples were stained with immunofluorescent antibodies after undergoing tension, and confocal laser microscopy revealed that RUNX2 in the mechanical tension treatment group showed a stronger fluorescence signal than that in the non‐treated group. This enhancement was most significant when mechanical traction was applied to PDLSCS for 24 h (Figure 2D). Therefore, in the follow‐up experiment, we explored the effect of 24‐h treatment on mitochondrial autophagy in PDLSCs and assessed its ability to mediate osteogenic differentiation.

### 3.3 Effects of CTS on mitochondrial autophagy in PDLSCs

**FIGURE 3:**
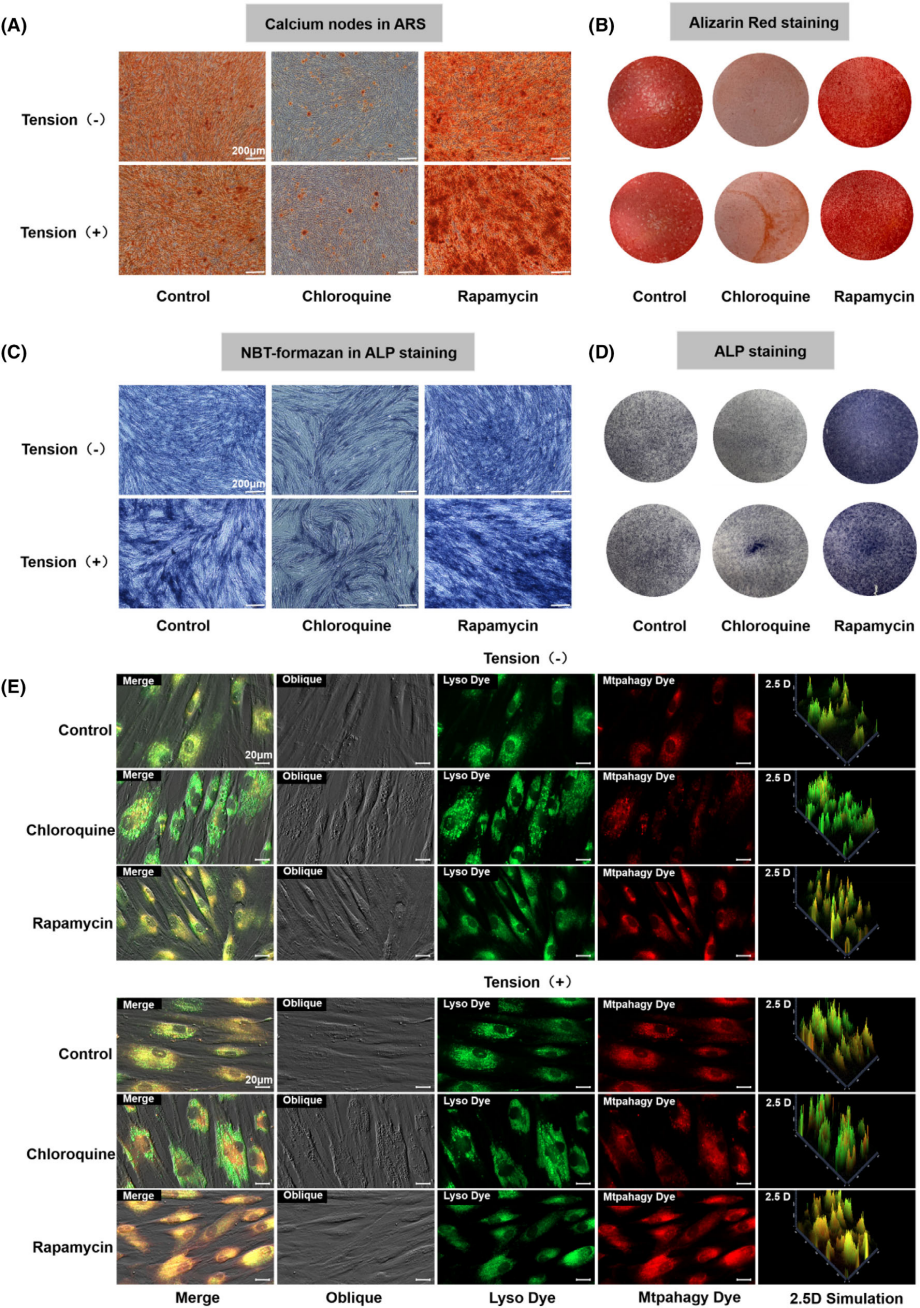
Effects of CTS on mitochondrial autophagy in PDLSCs. (A) Calcium nodules in alizarin red‐stained cells after osteogenic induction for 21 days. (B) Osteogenic differentiation was detected using alizarin red staining on day 21 (scale bars are 200 μm). (C) NBT formation in ALP‐stained cells after 7 days of osteogenic differentiation. (D) Osteogenic differentiation was detected using ALP staining on day 7 (scale bars are 200 μm). (E) The effect of CTS on mitochondrial autophagy was detected using the mitochondrial autophagy kit (scale bars are 20 μm; Lyso Dye: green; and Mtphagy Dye: red).

During the experiment, the autophagy inhibitor chloroquine and the autophagy inducer rapamycin were applied to alter mitochondrial autophagy, and the changes in mitochondrial apoptosis in PDLSCs after applying CTS and the relationship between these changes and PDLSC osteogenic differentiation were investigated. Mtphagy Dye is chemically bound to mitochondria in cells and emits weak fluorescence. When autophagy occurs, the injured mitochondria combine with the lysosome and the pH will decrease, becoming acidic. This change causes Mtphagy Dye to produce strong fluorescence. Mitochondrial autophagy detection by confocal microscopy showed that Mtphagy showed red fluorescence, the Lyso autophagic lysosome marker showed green fluorescence, and the two colours colocalized. The induction group was treated with 10 μmol/L autophagy inducer rapamycin for 8 h. Mitochondrial autophagy in PDLSCs was significantly enhanced, and the autophagy was significantly weakened after the addition of 5 μmol/L chloroquine for 8 h in the inhibition group. Moreover, mitochondrial autophagy in PDLSCs was significantly enhanced after applying CTS (Figure 3E). To further verify the relationship between mitochondrial autophagy and osteogenic differentiation in PDLSCs after applying CTS, we measured the activity of ALP, a marker of early osteogenic differentiation, with a BCIP/NBT alkaline phosphatase chromogenic kit 7 days after osteogenic induction differentiation. The ALP activity was upregulated in the rapamycin‐induced group, and more NBT formed, while the ALP activity was reduced in the chloroquine‐inhibited autophagy group. Compared with the group without CTS, the activity of ALP was significantly enhanced after CTS was applied, and more NBT was formed (Figure 3A,B). To further verify the relationship between mitochondrial autophagy and the osteogenic differentiation of PDLSCs after applying CTS, alizarin red was applied to assess the matrix mineralization after 15 days of osteogenic induction culture (Figure 3C,D). Calcium nodules are considered late osteogenic markers in DPLCs. The formation of calcium nodules increased in the rapamycin autophagy‐induced group and decreased in the chloroquine autophagy‐inhibited group. After CTS was applied, more calcium nodules were formed. These results and the results of mitochondrial autophagy detection confirmed that mitochondrial autophagy positively correlated with CTS in promoting PDLSC osteogenic differentiation. Mitochondrial autophagy and osteogenic differentiation of PDLSCs can be promoted by applying CTS.

### 3.4 Detection of osteogenic differentiation‐specific gene and protein expression after applying CTS

**FIGURE 4:**
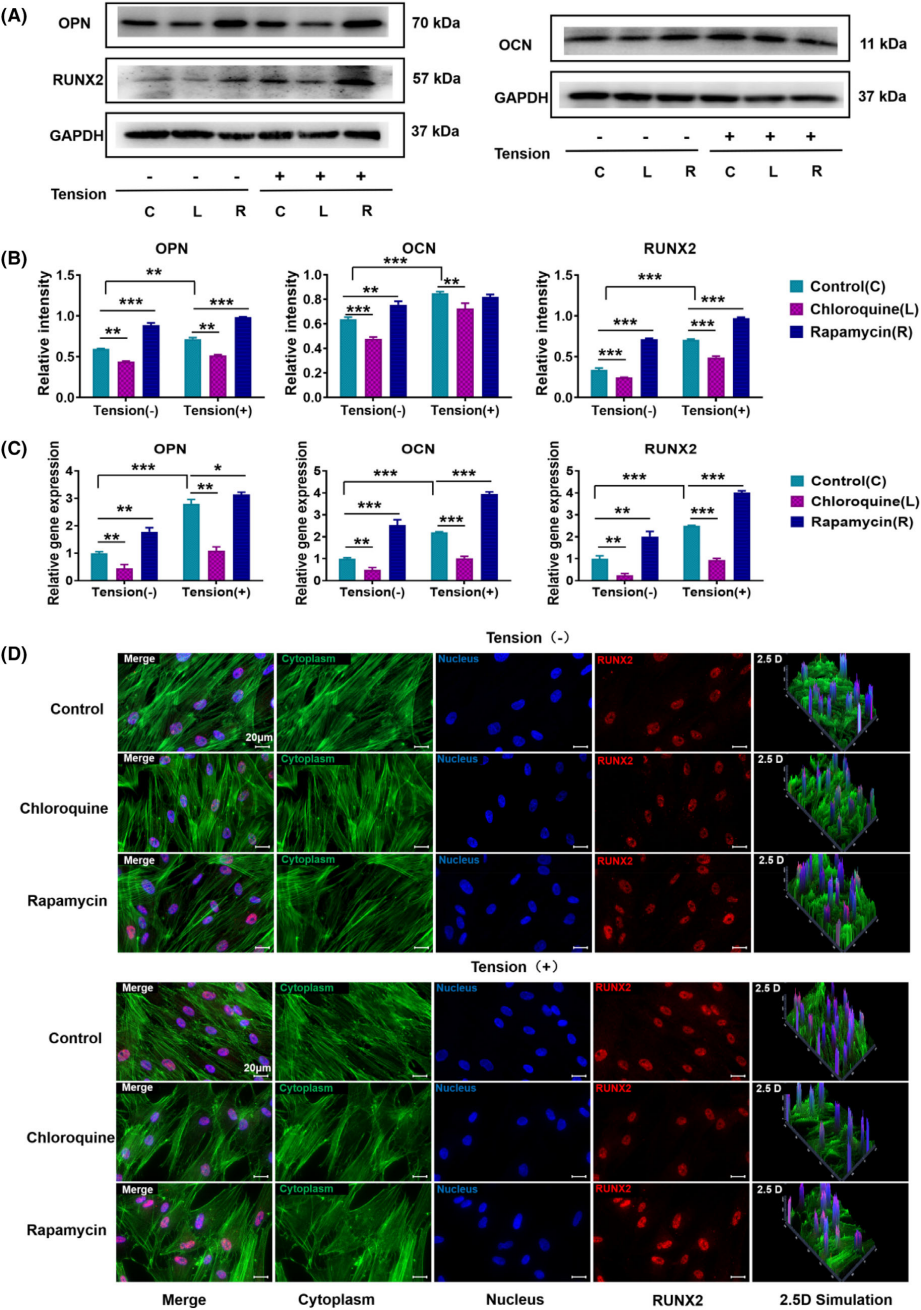
Detection of osteogenic differentiation‐specific gene and protein expression after applying CTS. (A) The expression levels of OPN, OCN, and RUNX2 were assessed using Western blotting after pretreatment with the autophagy inducer rapamycin or the autophagy inhibitor chloroquine and subsequent application of CTS for 24 h (GAPDH was used as the internal control). (B) Quantification of OCN, OPN, and RUNX2 expression levels. GAPDH levels were set as the internal normalized control. Student's *t* test was used for the statistical analysis. Data are presented as the mean ± SD (*n* = 4). Statistical analysis: ** *p* < 0.01 and *** *p* < 0.001. (C) RT‐PCR was used to detect the gene expression levels of OCN, OPN, and RUNX2 in PDLSCs after pretreatment with rapamycin or chloroquine and the subsequent application of CTS for 24 h. GAPDH levels were set as the internal normalized control. Student's *t* test was used for the statistical analysis. Data are presented as the mean ± SD (*n* = 4). Statistical analysis: ** *p* < 0.01 and *** *p* < 0.001. (D) Immunofluorescent micrographs of treated PDLSCs (scale bars are 50 μm; cytoplasm: green; nucleus: blue; and RUNX2: red).

To further confirm the influence of CTS on PDLSCs, we assessed the the gene expression of osteogenic markers by RT‐PCR in different treatment groups 7 days after osteogenic induction differentiation. The expression of RUNX2, OCN and OPN was upregulated in the rapamycin autophagy induction group, while the expression of these genes was downregulated in the chloroquine autophagy inhibition group (Figure 4C). After applying CTS, the expression levels of OPN, a marker of advanced osteogenic differentiation, were 2.79 times higher than those in the non‐CTS group, and the expression of RUNX2, a key transcription factor encoding osteogenic differentiation, was 2.48 times higher than that in the non‐CTS group. Additionally, the expression of OCN, a bone tissue‐specific protein, was 2.21 times higher than that in the CTS group.

In addition to detecting the mRNA expression of osteogenic differentiation in PDLSCs, we measured the osteogenic markers proteins using Western blotting and immunofluorescence. After CTS, the immunofluorescence staining was observed by confocal microscopy, and the fluorescence signal of RUNX2 in the rapamycin autophagy induction group was stronger than that in the control group, while the signal was weaker in the chloroquine autophagy inhibition group. RUNX2 showed a stronger signal when CTS was applied (Figure 4D). Western blotting (Figure 4A,B) revealed that the osteogenic markers OPN, OCN and RUNX2 were significantly enhanced after applying CTS. This conclusion was further confirmed through the statistical analysis (OCN levels increased 1.33 times, OPN 1.21 times and RUNX2 2.07 times). In conclusion, these findings showed that applying CTS can promote osteogenic differentiation of PDLSCs. The expression of osteogenic markers was significantly enhanced in the CTS‐treated PDLCs. Moreover, the addition of a mitochondrial autophagy inducer can up‐regulate the expression of the above proteins and genes, while the addition of an autophagy inhibitor can reduce the expression of related genes and proteins.

### 3.5 Detection of autophagy‐related gene protein expression after application of CTS

**FIGURE 5:**
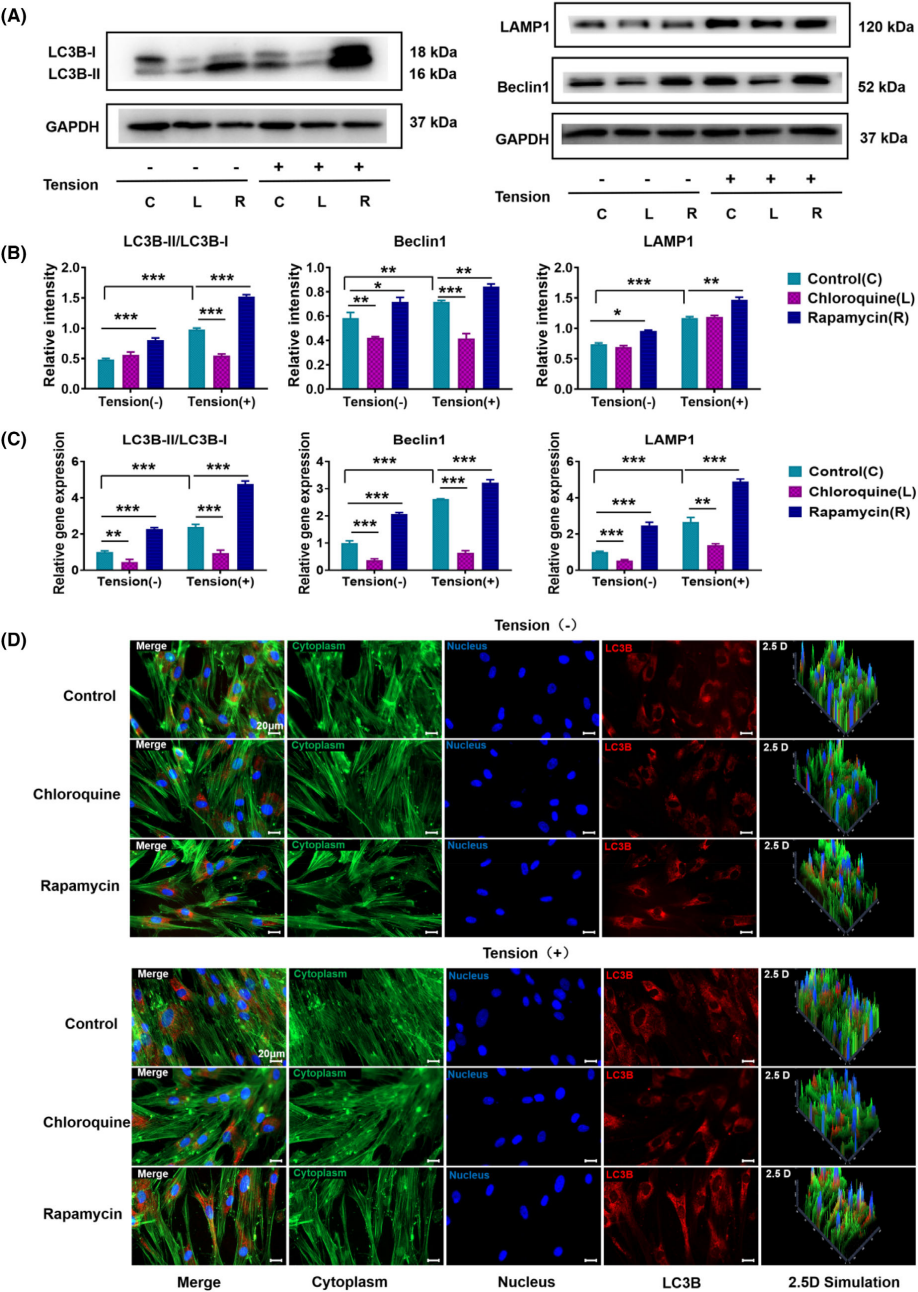
Detection of autophagy‐related gene protein expression after applying CTS. A) The expression levels of LC3B, LAMP1, and Beclin1 were detected using Western blotting after pretreatment with the autophagy inducer rapamycin or the autophagy inhibitor chloroquine and the subsequent application of CTS for 24 h. GAPDH was used as the internal control. B) Quantification of LC3B, LAMP1, and Beclin1 expression levels. GAPDH levels were set as the internal normalized control. Student's *t* test was used for the statistical analysis. Data are presented as the mean ± SD (*n* = 4). Statistical analysis: ** *p* < 0.01 and *** *p* < 0.001. (C) RT‐PCR was used to measure the gene expression levels of LC3B, LAMP1, and Beclin1 in PDLSCs after pretreatment with autophagy rapamycin or chloroquine and the subsequent application of CTS for 24 h. GAPDH levels were set as the internal normalized control. Student's *t* test was used for the statistical analysis. Data are presented as the mean ± SD (*n* = 4). Statistical analysis: ** *p* < 0.01 and *** *p* < 0.001. (D) Immunofluorescent micrographs of treated PDLSCs (scale bars are 50 μm; cytoplasm: green; nucleus: blue; and LC3B: red).

LC3B is the most critical signature protein in the autophagy signalling pathway and comprises 125 amino acid residues. After protein synthesis, C‐terminal glycine is exposed, yielding the cytoplasmic form LC3B‐I. During autophagy, the glycine exposed at the C‐terminal undergoes a process similar to ubiquitination and becomes LC3B‐II, which is located on the inner and outer membranes of autophagosomes. LCB localization information can be used as a common marker of autophagosomes to assess autophagosome development and changes. In this experiment, to explore the changes in mitochondrial autophagy in PDLSCs after applying CTS and the relationship between these changes and the osteogenic differentiation of PDLSCs, the protein expression of autophagy‐related markers were detected using Western blotting and immunofluorescence. The ratio of LC3B‐II/LC3B‐I, Beclin1 and LAMP1 in the rapamycin autophagy induction group was clearly enhanced compared with that in the control group, while that in the chloroquine autophagy inhibition group was decreased (Figure 5A,B). The protein expression of the above autophagy‐related markers was significantly higher in the CTS group than in the non‐CTS group. The statistical analysis showed that after applying CTS, the ratio of LC3B‐II/LC3B‐I, a key signature protein of the autophagy signalling pathway, was increased to 1.67 times that of the group without CTS. Furthermore, the expression of Beclin1 increased to 1.22 times that of the group without CTS, and the expression of LAMP1 increased to 1.58 times that of the control group. The immunofluorescence results were consistent with the Western blotting results. LC3B in the rapamycin autophagy induction group showed a stronger fluorescence signal than that of the control group, while the signal in the chloroquine autophagy inhibition group was weaker. LC3B in the group with CTS applied showed a stronger signal than that in the group without CTS (Figure 5E). In addition, the expression mRNA corresponding to the autophagy‐related proteins were measured using RT‐PCR. The ratio of LC3B‐II/LC3B‐I and the mRNA expression of Beclin1 and LAMP1 were 2.38, 2.61 and 2.66 times higher in the CTS group than those in the non‐CTS group (Figure 5C). In conclusion, applying CTS can promote the osteogenic differentiation of PDLSCs by inducing mitochondrial autophagy.

## 4 DISCUSSION

Orthodontic tooth movement is achieved by applying static pressure and tension on both sides of the tooth requiring orthodontic treatment to achieve tooth displacement. This process is accompanied by decreased osteogenic activity on the pressure side and increased osteogenic activity on the tension side.^22^ Shen et al. found that periodic tension promotes the osteogenic differentiation of PDLSCs.^9^, ^23^ Yang et al. found that periodic tension promotes PDLSC osteogenic differentiation by activating the YAP pathway.^24^ Thus, the mechanism underlying the change in PDLSC osteogenic differentiation mediated by stretch tension requires further study. Recent studies have revealed that the development and differentiation of mesenchymal stem cells are closely related to the fusion and division of mitochondria, and the microenvironment can affect the autophagy level of mesenchymal stem cells.^25^, ^26^, ^27^, ^28^, ^29^ Mitochondrial autophagy is a special form of mitochondrial quality control considered an important cell survival mechanism responsible for removing damaged, redundant, or aging mitochondria. Nollet et al. found that autophagy is related to osteoblast mineralization and bone homeostasis after the knockout of an essential autophagy gene in osteoblast‐specific autophagy‐deficient mice.^30^ In the process of osteogenic differentiation induced by stem cells, the autophagy inducer rapamycin is used to improve mitochondrial autophagy, resulting in changes in mitochondrial morphology and studies have demonstrated that mitochondrial division is proportional to mitochondrial autophagy.^31^ Additionally, rapamycin promotes the mitochondrial autophagy pathway, thus promoting the osteogenic differentiation of stem cells. The regulation of intracellular mitochondrial content is a potential mechanism by which autophagy regulates the early targeted differentiation of stem cells, and mitochondrial autophagy may be a potential therapeutic target in bone‐related diseases.^32^, ^33^, ^34^

Therefore, this study focused on exploring the changes in the osteogenic differentiation ability of PDLSCs under CTS. The autophagy process was affected by the addition of the autophagy inhibitor chloroquine and the autophagy inducer rapamycin, and the changes in mitochondrial apoptosis in PDLSCs after applying CTS and the relationship between these changes and osteogenic differentiation were discussed. Chloroquine inhibits the late stage of autophagy, that is, the fusion of the autophagosome and the lysosome.^35^, ^36^, ^37^, ^38^ Blocking this fusion inhibits autophagy at the autophagosome stage, thus inhibiting mitochondrial autophagy and the osteogenic differentiation of mesenchymal stem cells. Rapamycin inhibits the autophagy signalling pathway mTOR, promotes the fusion of autophagosomes and lysosomes, increases the number of autophagosomes, degrades cell contents and promotes cell metabolism and self‐renewal, thus promoting the osteogenic differentiation of bone marrow mesenchymal stem cells.^39^, ^40^ In this experiment, alkaline phosphatase staining and alizarine red staining results showed that after 7 days of osteogenic differentiation, compared with that in the group without CTS, the activity of ALP was significantly enhanced in the CTS group, and more NBT was formed; after 15 days, more calcium nodules were formed in the CTS group. Moreover, applying CTS can effectively increase the expression levels of OPN, OCN and RUNX2, key growth factors related to osteogenic differentiation. This increased expression improves the activity and mineralization ability of PDLSC alkaline phosphatase and promotes the osteogenic differentiation of mesenchymal stem cells. To further verify the relationship between CTS and osteogenic differentiation promotion in PDLSCs and mitochondrial autophagy, we measured the expression levels of LC3B, the most critical signature protein in the autophagy signalling pathway that plays a role in the extension‐closure mechanism of autophagosomes. LC3B exists in two forms, one of which does not bind to lipids and is widely distributed in the cytoplasm (LC3B‐I). LC3B‐II binds lipid phosphatidyl ethanolamine at the two membranes of mature autophagosomes. During autophagosome formation, homotype LC3B‐I is transformed into LC3B‐II and can be used as a marker for the number of autophagosomes and the induction of autophagy.^41^ Here, it was found that the expression levels of LC3BII and LC3BII/LC3BI increased after applying CTS. Meanwhile, the expression of osteogenic proteins ALP, OPN, OCN and RUNX2 also showed this trend. The results of this study suggest that mitochondrial autophagy is positively correlated with the osteogenic differentiation of PDLSCs after applying CTS, and CTS can induce mitochondrial autophagy, thus promoting the osteogenic differentiation of PDLSCs.

This study preliminarily demonstrated the promoting effect of stretch tension on the osteogenic differentiation ability of PDLSCs, which may be realized through the promotion of mitochondrial autophagy. This finding provides new insights into the mechanism of increased osteogenic differentiation on the tension side of orthodontic teeth and new experimental evidence for the involvement of mitochondrial autophagy in the regulation of osteogenic differentiation.

## AUTHOR CONTRIBUTIONS

All authors contributed to the study concept and design. Xiaoru Shao and Huiqin Su conducted the in vitro experiments on PDLSCs. Zhong Hu collected the data. Xiaoru Shao performed the analysis and drafted the manuscript. Yunfeng Lin and Yuzhong Wang initiated the research, designed research studies, analysed data and acquired funding. All authors have reviewed and approved the final version of the manuscript.

## FUNDING INFORMATION

This work was supported by grants from the National Natural Science Foundation of China (Grant Nos. 82100984, 82370929, 81970916), Postdoctoral Innovation Project of Shandong Province (Grant Nos. SDCX‐ZG‐202202007), Postdoctoral Program of Affiliated Hospital of Jining Medical University (Grant Nos. JYFY303575) and PhD Research Foundation of Affiliated Hospital of Jining Medical University (Grant Nos. 2020‐BS‐002), National Key R&D Program of China (2019YFA0110600), Sichuan Science and Technology Program (2022NSFSC0002), Sichuan Province Youth Science and Technology Innovation Team (2022JDTD0021), Research and Develop Program, West China Hospital of Stomatology Sichuan University (RD03202302).

## CONFLICT OF INTEREST STATEMENT

The authors declare no competing interests.

## Data Availability

The data that support the findings of this study are available from the corresponding author upon reasonable request.
